# Supplementary material for: The interactome of intact mitochondria by cross-linking mass spectrometry provides evidence for coexisting respiratory supercomplexes
Source: Mol Cell Proteomics. 2017 Dec 8;17(2):216–32. doi: 10.1074/mcp.RA117.000470 (PMC5795388; doi:10.1074/mcp.RA117.000470)
Supplement: Supplemental Data [file supp_RA117.000470_133922_0_supp_23978_fzffwf.zip › spectra_annotation/mito_DR_spectra_annotation/35-1-13-1-28-1.pdf]

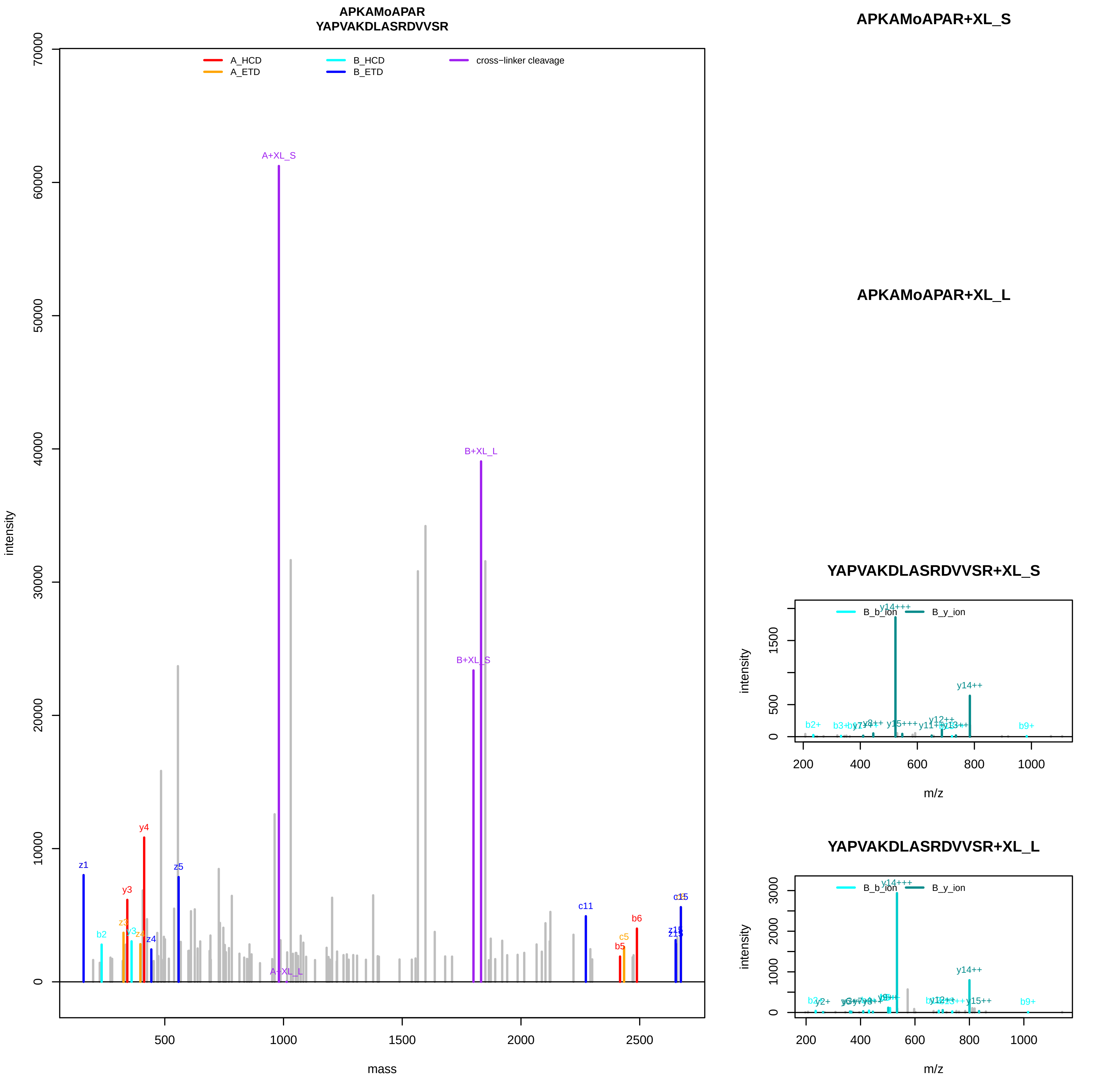
APKAMoAPAR
YAPVAKDLASRDVVSR
A_HCD
A_ETD
B_HCD
B_ETD
cross−linker cleavage
A+XL_S
B+XL_L
B+XL_S
A+XL_L
z1
b2
z3
y3
y4
z4
z5
c11
c5
b5
b6
c15
z15
z13
intensity
mass
0
10000
20000
30000
40000
50000
60000
70000
500
1000
1500
2000
2500
APKAMoAPAR+XL_S
APKAMoAPAR+XL_L
YAPVAKDLASRDVVSR+XL_S
B_b_ion
B_y_ion
y14+++
y14++
b2+
b9+
y15+++
y12++
intensity
m/z
YAPVAKDLASRDVVSR+XL_L
y14+++
y14++
y2+
y15++
b9+
200
400
600
800
1000
